# Acceptability and feasibility of the Ushirikiano treatment model for Kenyan adolescents: a pilot randomized controlled trial

**DOI:** 10.3389/frcha.2026.1890788

**Published:** 2026-09-03

**Authors:** Nabila Amin Ali, Shillah Mwaniga Mwavua, Obadia Yator, Joseph Kathono, Anja C. Huizink, Manasi Kumar

**Affiliations:** 1 Department of Clinical, Neuro- and Developmental Psychology, Vrije Universiteit Amsterdam, Amsterdam, Netherlands; 2 Child and Adolescent Mental Health Unit, Mathari National Teaching and Referral Hospital, Nairobi, Kenya; 3 Department of Health, Nutrition and Wellness, Nairobi, Kenya; 4 Institute of Tropical and Infectious Diseases, University of Nairobi, Nairobi, Kenya; 5 Department of Psychology, University of Gothenburg, Gothenburg, Sweden; 6 Department of Population Health, Institute for Excellence in Health Equity, New York University Grossman School of Medicine, New York, NY, United States

**Keywords:** adolescents, depression, fluoxetine, group interpersonal psychotherapy, Kenya, primary care

## Abstract

**Introduction:**

Adolescents in low- and middle-income countries experience high rates of common mental disorders (CMDs) but have limited access to adolescent-friendly, evidence-based care. While Interpersonal Psychotherapy for Adolescents (IPT-A) is effective, brief group IPT-A delivered through task-shifted, stepped-care has not been rigorously evaluated in Kenyan primary care.

**Methods:**

We conducted a pilot randomized controlled trial to examine the acceptability, feasibility, and preliminary effectiveness of the Ushirikiano treatment model, a task-shifted, stepped-care approach combining a brief (four-session) group IPT-A (IPT-G-A) delivered by community health promoters, with fluoxetine for severe cases where indicated. Adolescents aged 12–18 years with DSM-5 TR depression, anxiety, and/or somatic symptom disorder (*N* = 32) were randomized to the Ushirikiano Model or enhanced treatment as usual (eTAU). Quantitative and qualitative data were collected at baseline, post-treatment, and at follow-up at months 1 and 2. The COM-B framework informed the assessment of behavior change.

**Results:**

All adolescents in the intervention arm were retained compared with 69% in eTAU. Intervention fidelity ranged from moderate to high and showed improvement with supervision. Participants found the intervention acceptable, relevant, and supportive, and reported applying IPT-G-A skills to handle interpersonal and school-related stressors. Community health promoters reported increased confidence with blended training and ongoing supervision. Preliminary outcomes trends suggested potential reductions in depressive symptoms and modest functional improvement in the Ushirikiano arm, with most participants achieving symptomatic remission at the month 2 follow-up. As a pilot study, these findings provide preliminary evidence of potential benefit and support further evaluation of the intervention in a fully powered trial.

**Conclusion:**

The Ushirikiano treatment model appears acceptable and feasible for addressing adolescent depression in Kenyan primary care. While preliminary findings suggest the potential for improvements in depressive symptoms and functioning, the study was not designed to establish effectiveness and did not assess for anxiety and somatic symptom disorder. These findings support further evaluation in fully powered trials to determine clinical effectiveness for CMDs, cost-effectiveness, and optimal implementation strategies.

**Clinical Trial registration:**

https://pactr.samrc.ac.za/TrialDisplay.aspx?TrialID=33227, Pan African Clinical Trial Registry, PACTR202503728947243.

## 1 Introduction

Depression, anxiety, and behavioral disorders are major causes of illness and disability, with suicide ranking as the third leading cause of death among people aged 15–29 (1). A substantial proportion of mental illnesses begins during adolescence, with as many as one in three onsets of every disorder emerging before age 14 and nearly one in two by age 18 (2). Globally, approximately 14% of children and adolescents aged 10–19 years' experience mental conditions, many of which remain untreated (3, 4). The Mental Health Gap Action Program (mhGAP), developed by the World Health Organization (WHO), aims to bridge this treatment gap by providing evidence-based guidance for recognizing and managing mental, neurological, and substance use disorders in primary health care settings (5). The mhGAP Intervention Guide advocates a stepped-care model that emphasizes psychosocial interventions, such as psychoeducation, problem-solving counseling, cognitive-behavioral therapy, and interpersonal therapy (IPT), as the primary treatments for adolescents with emotional disorders. Pharmacological treatments, such as fluoxetine, are generally reserved for moderate to severe depression and typically administered under specialist supervision (6).

IPT, as one of the recommended interventions by WHO, was originally developed to treat depression among adults in the 1970s (7, 8). It is a structured time-limited psychotherapy (12–16 sessions) initially delivered individually by mental health specialists (7, 8). Ever since IPT has been adapted to treat various conditions, different age groups, special populations, and in group settings (7, 9–12) In 2003 Group IPT was evaluated among adults in sub-Saharan Africa through a cluster-randomized trial in rural Uganda, where lay-delivered group IPT substantially reduced depression and dysfunction relative to usual care, providing early evidence of feasibility for this task-shifted approach in the region (13). In 2019, the WHO introduced group IPT, delivered by non-mental health workers in primary care settings, to facilitate task shifting in low- and middle-income countries (LMICs). This method seeks to enhance the intervention's accessibility and practicality (14).

Most public health facilities in Kenya lack adolescent-friendly mental health services, and there is a critical shortage of mental health specialists (15, 16). The Kenyan Ministry of Health has adapted the mhGAP principles into its national clinical guidelines for the management of common mental disorders (17). These guidelines focus on integrating mental health services into primary healthcare, supporting early detection, psychosocial treatment, and proper referral pathways. They also encourage task-sharing models that allow non-specialist providers, such as primary care workers and community health promoters, to deliver evidence-based mental health care for adolescents (17). This strategy seeks to close the mental health treatment gap and enhance access to mental health services in settings with limited resources.

Existing IPT research in Kenya has largely focused on adults and adolescent mothers, with minimal inclusion of male adolescents or evaluations of the brief, 4-session group IPT for adolescents (IPT-G-A) (18–20). Interpersonal Psychotherapy for Adolescents (IPT-A) was adapted from the adult version by Laura Mufson as a time-limited, 12-session, individualized weekly intervention to treat depression in adolescents (21–23). IPT-A improves depressive symptoms and interpersonal functioning by teaching adolescents better communication and interpersonal skills (21). This current research favors the brief 4-session adapted IPT-A group treatment over individual therapy for various reasons: its scalability, aligning with the task shifting approach, cost effectiveness, and ability to reach more adolescents in a shorter time (10, 21, 24, 25). The 4 sessions were guided by the WHO group IPT manual, which outlines how to adapt the 8-session group IPT to a brief 4-session format (14). For both approaches, there is a pre-group session that allows facilitators to engage with each participant individually. The briefer version combines assessment, formulation, and initial intervention in the first session. The middle phase includes sessions 2 and 3, during which problem areas are actively addressed. Finally, the termination phase (session 4) involves consolidation and closure. The group format also enhances peer support, reduces stigma, and improves engagement, while the brief structure increases feasibility and retention, considering the dynamics with this special age group (14). Group IPT-A has also been evaluated in mixed-gender, general adolescent populations elsewhere in sub-Saharan Africa; a cluster-randomized trial among orphaned and vulnerable adolescents in South Africa found that group IPT reduced depressive symptoms relative to standard care, indicating that this approach extends beyond the peripartum and maternal samples that dominate the Kenyan literature (26).

*The Ushirikiano treatment model*, developed by the lead researcher with a background in psychiatry, employs a stepped collaborative care approach based on the WHO mhGAP guideline. It combines brief group (4 group sessions) Interpersonal Psychotherapy for Adolescents (IPT-G-A), delivered by community health promoters to address common mental disorders (CMD), with the use of antidepressant (Fluoxetine) medication as part of integrated primary care services. *This pilot randomized controlled trial aimed to assess the acceptability and feasibility of the Ushirikiano treatment model and to explore its preliminary effectiveness for adolescents with CMDs in Nairobi, Kenya. In line with the WHO mhGAP guideline recommendations on task-shifted, integrated mental health care* (6) *and the Kenyan Ministry of Health national clinical guidelines on the management of common mental disorders, there is an urgent need for scalable, task-shifted interventions* (3)*.*

**Figure 1:**
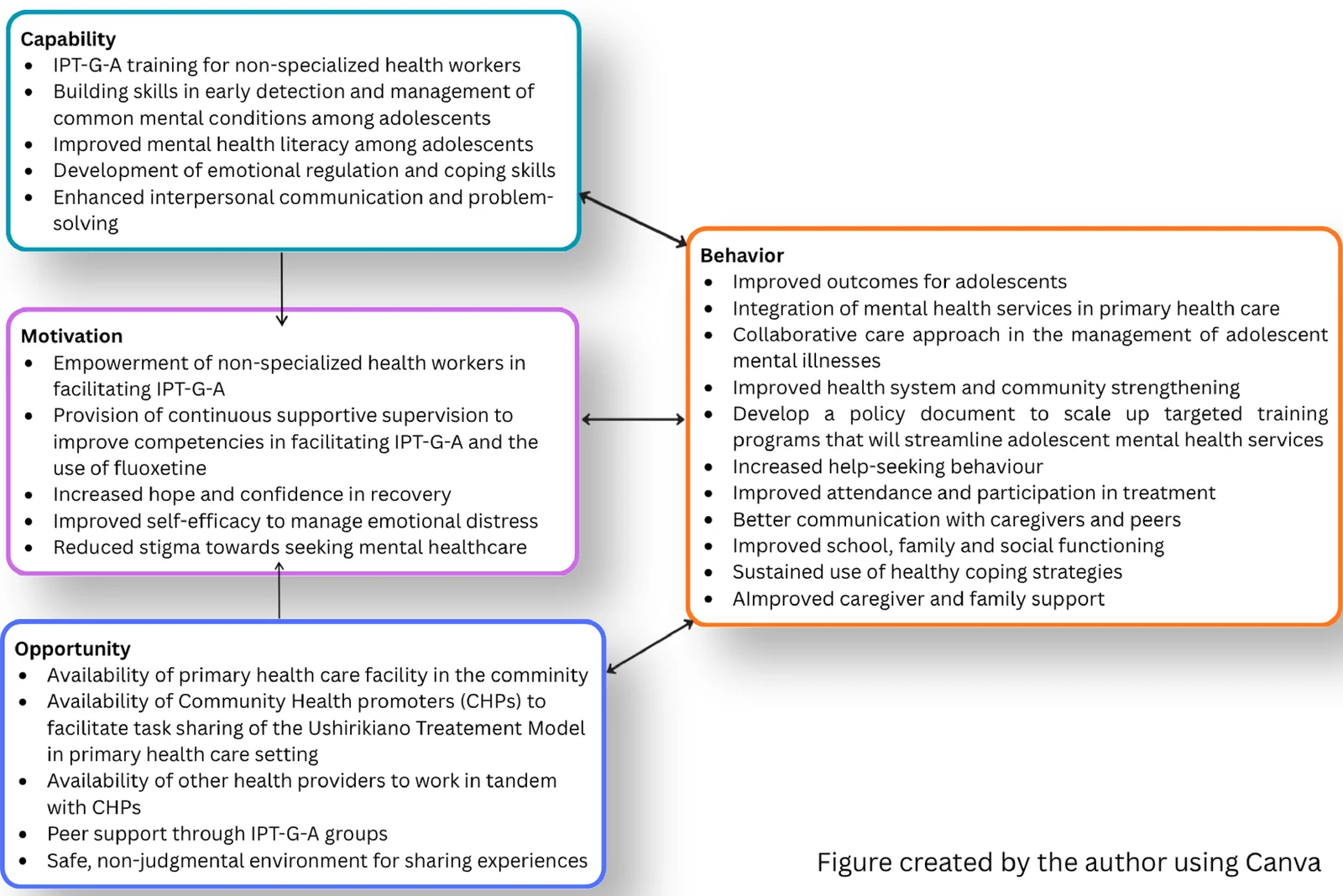
The COM-B model to understand behavior change using the Ushirikiano model.

The acceptability and feasibility of the *Ushirikiano Treatment Model* were evaluated using the *Capability, Opportunity, Motivation to Behaviour (COM-B) model* (Figure 1), also known as the Behaviour Change Wheel (BCW). It conceptualizes behavior as the interaction among capability (an individual's capacity to engage in behavior modification), opportunity (environmental factors that influence individual behavior), and motivation (the willingness to change). This model will guide the understanding of behavior change due to the Ushirikiano treatment model by non-specialized health workers for Kenyan adolescents with common mental disorders (CMD; DSM-5-TR Major depressive disorder, Anxiety disorder, and Somatic symptom disorder (27). These 3 components of the COM-B model are further divided into six subdomains: Capacity (Physical and Psychological), Opportunity (Physical and Social), and Motivation (Reflective and Automatic) (28, 29). Using a behavior-based theoretical model is essential because interventions grounded in behavioral frameworks have been shown to be more effective than those without a theoretical basis (30).

## 2 Materials and methods

### 2.1 Design and setting

This pilot randomized controlled trial was carried out at a single public primary care facility in Nairobi and embedded within routine services using a stepped collaborative care framework. IPT-G-A groups were delivered during school closure, with a 2-month follow-up after school reopening to assess symptom change and skill generalization during that period.

### 2.2 Participant

Adolescents aged 12–18 years attending outpatient clinics or identified through community mobilization were systematically screened. Inclusion criteria were DSM-5-TR diagnoses of depression, anxiety, and/or somatic symptom disorder and willingness to participate; adolescents with suicidal ideation or recent attempts were included and managed within stepped care. Exclusion criteria included other primary psychiatric diagnoses, serious medical illness, poor response to fluoxetine, or current antipsychotic use. Community Health Promoters (CHPs) from the facility were recruited willingly and trained to deliver the intervention. CHPs received blended training (virtual and in-person), with ongoing supervision and fidelity monitoring.

### 2.3 Randomization and procedures

Following assent and caregiver consent, eligible adolescents were screened with the DSM-5 Level 1 Cross-Cutting Symptom Measure, and those who scored high on the CMDs were administered the Kiddie Schedule for Affective Disorder and Schizophrenia (KSADS-COMP), which was digitized using the Research Electronic Data Capture (REDCap) tool hosted by the University of Nairobi (31). Participants' diagnostic profile, symptom severity, and gender were reviewed to characterize the study population before the random allocation in a 1:1 ratio either to the intervention arm or control arm. Particular attention was given to achieving comparable representation of male and female participants across study arms.

An independent clinician, not involved in recruiting, baseline assessments, or delivering the intervention, created the allocation schedule in Microsoft Excel. Allocation assignments were concealed until the baseline assessments for participants, who would form an 8-person group for the intervention and eTAU, were completed. Subsequently, participants were assigned to either the intervention or control group. We then created two groups of 8 participants each, one for the intervention and one for eTAU; a total of 32 participants were included in the pilot study.

### 2.4 Intervention (Ushirikiano treatment model)

The Ushirikiano treatment model comprised three components: (1) stepped collaborative care with systematic screening, stratification, and referral pathways; (2) brief group IPT-A consisting of one individual pre-group session and four weekly 90 min group sessions delivered by trained CHPs for all participants; and (3) fluoxetine augmentation for participants who had severe depression with suicidal ideation and or attempts, at the discretion of the psychiatrist in collaboration with clinicians in the study, and monitored in primary care by the CHPs supported by the whole team. Only 2 participants qualified to be given Fluoxetine after consultative discussion with the research team; the rest of the adolescents with severe depression were monitored closely as the intervention continued.

Due to the nature of the intervention, blinding of participants and facilitators, along with the research team, was not feasible. Several measures were implemented to minimize contamination between study arms. Participants assigned to the intervention and control conditions attended study activities on separate days and were encouraged to maintain confidentiality regarding study procedures and intervention content. Facilitators signed a confidentiality and contamination mitigation agreement prior to study implementation and received guidance on maintaining separation between study conditions. Confidentiality was reinforced throughout intervention delivery, and participants were periodically asked about communication regarding intervention content with other study participants as part of contamination monitoring procedures.

### 2.5 Enhanced treatment as usual (eTAU)

Participants in the eTAU group received a single group session as an enhancement to the treatment as usual; this session is not part of the standard services offered at the facility. The only social worker who sees adolescents with mental illness at the facility took the participants through this group session; areas of discussion included peer pressure, substance use, school and education, and challenges they face as adolescents. Typically, the facility provides mental health care when adolescents present with symptoms; the care provided is not structured but mainly includes psychoeducation, individual and group therapy sessions, with occasional medication. However, medication is not always available due to shortages of both drugs and visiting clinical officers. Patients who cannot be managed at the facility are usually referred to a nearby Non-Governmental Organization that supports youth well-being, including mental health services. In severe cases of mental illness, adolescents are either transferred to a nearby county hospital or a national mental health facility.

### 2.6 Data collection and outcomes

A mixed-methods approach was used to evaluate clinical outcomes, acceptability, feasibility, and fidelity. Quantitative data were gathered using standardized tools such as the PHQ-9 and WHO-DAS 2.0 at baseline, mid-intervention, post-treatment, and at two-month follow-up. Key informant interviews, focus group discussions, and observations by the research team were used to assess perceived impact and implementation experiences. Fidelity was monitored via structured supervision tools, researcher/supervisor observations, and session logs (Table 1).

**Table 1:** Outcome measures and assessment schedule.

Outcome Domains	Measure and Purpose of Assessment	Assessment Time Points
Eligibility/Diagnosis	DSM-5 Level 1 Cross-Cutting Symptom Measure; KSADS-COMP to screen and confirm DSM-5 TR diagnoses prior to randomization	Baseline (Week 0)
Primary Clinical Outcome	Patient Health Questionnaire-9 (PHQ-9) to score depressive symptom severity and change over time	Baseline (IPT-G-A & eTAU); Weekly during intervention (weeks 1–4); midpoint (week 2) for eTAU, 2-months follow-up (IPT-G-A & eTAU)
Functional Outcome	WHO Disability Assessment Schedule 2.0 (WHO-DAS 2.0) to measure functional impairment and improvement	Baseline; Post-intervention (week 4); 2-months follow-up for both IPT-G-A & eTAU
Intervention Fidelity	WHO Group IPT Task Checklist to assess adherence to IPT-G-A structure and core therapeutic processes	Pre-group session; each group session (4 sessions)
Acceptability & Feasibility (Qualitative)	Capacity, Opportunity, Motivation to Behavior (COM-B) Model Semi-structured interviews and focus groups (participants & facilitators) Perceived relevance, barriers, and implementation experience	During Intervention, Post-intervention (week 4), and 2-months follow-up for both IPT-G-A & eTAU

### 2.7 Data analysis

As a pilot study, it was not powered for definitive efficacy testing; analyses focused on descriptive statistics, patterns of symptom and functional change over time, and qualitative themes related to acceptability and feasibility. Group differences were examined descriptively, focusing on trajectories rather than formal hypothesis testing. Quantitative data were collected and managed using REDCap (Research Electronic Data Capture), and Microsoft Excel was used for descriptive analysis. Given the small sample size (*n* = 32) and the descriptive aim of this pilot, formal effect sizes and confidence intervals were not computed; raw between-arm differences in means, proportions, and PHQ-9/WHO-DAS 2.0 scores are reported in Table 2 and Figures 3, 4 and will be used to inform effect size and sample size calculations for the planned fully powered trial.

### 2.8 Ethical approvals and ethical considerations

Ethical approval was granted by the Kenyatta National Hospital/University of Nairobi Ethics & Research Committee (Approval no: P479/06/2024), Nairobi County, as well as the National Commission for Science, Technology, and Innovation (License no: NACOSTI/P/24/41938), and the Nairobi City County Government (Ref: NCCG/HWN/REC/693). Approval from all participating facilities was also obtained. Written informed consent was secured from parents or legal guardians, along with written assent from adolescents, before enrollment. Health professionals involved in the study also provided written consent. Participation was voluntary, and individuals could withdraw at any point without repercussions or impact on services. All data were kept confidential and used exclusively for research purposes.

### 2.9 Health care worker characteristics

Twelve health care workers participated in the pilot, including 7 community health promoters, 1 social worker, 2 psychologists, and 2 IPT supervisors. Health workers' ages ranged from 22 to 60 years, with diverse educational backgrounds, including vocational training and bachelor's degrees. Most were based at the Dandora II Health Center. Two previously trained IPT facilitators took part. Some trained CHPs did not participate due to competing commitments, missed significant training components, or due to misunderstandings that were later resolved.

### 2.10 Participant characteristics

**Figure 2:**
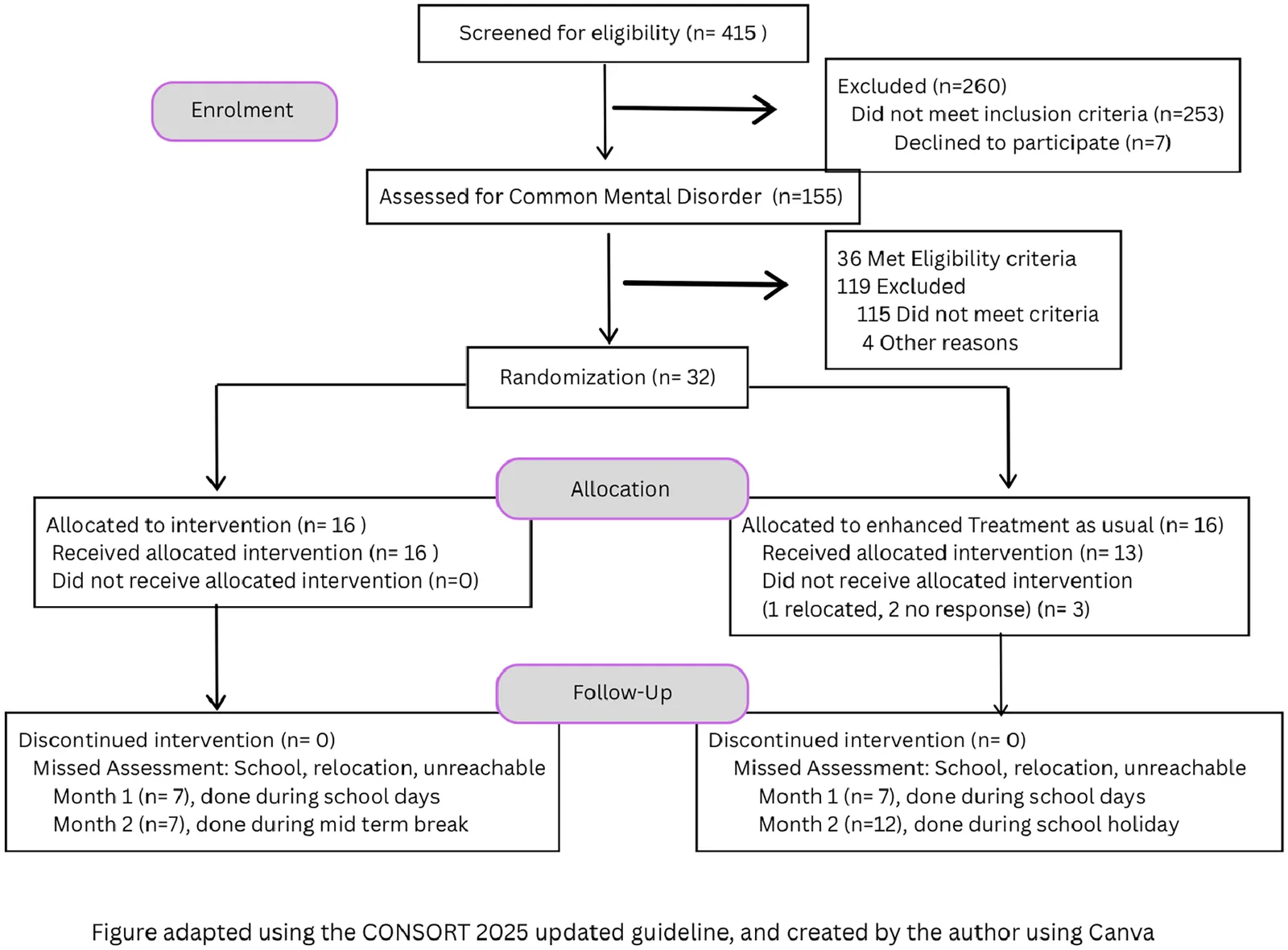
CONSORT flow diagram of participant recruitment, allocation, and follow-up (31).

A total of 155 adolescents aged 12–18 years were screened (82 males, 73 females) from among 415 adolescents approached (Figure 2). Thirty-six met criteria for one or more common mental disorders (CMDs), most commonly major depressive disorder (MDD), often with comorbid somatic symptom disorder or anxiety. Severe depression and suicidal ideation were frequent among those with MDD. Thirty-two adolescents met full inclusion criteria and were randomized (16 Ushirikiano; 16 enhanced treatment as usual; eTAU) (Figure 2). Intervention groups were mixed-gender and included early, mid, and late adolescents with varied symptom presentations.

## 3 Results

### 3.1 Facilitator fidelity and supervision

Structural fidelity was high across the pilot implementation, with delivery of one pre-group session, four 90-minute IPT-G-A sessions, and completion of planned follow-ups. Fidelity was evaluated using the WHO Group IPT Task Checklist for facilitators and supervisors, as outlined in the WHO Group IPT guidelines. This checklist assessed adherence to session structure, therapeutic process, and delivery competencies. Trained IPT supervisors independently completed fidelity checklists after directly observing sessions from the pregroup to the termination phase of the four IPT-A groups, including structured post-session review.

Each competency item was classified according to the WHO Group IPT Task Checklist for facilitators and supervisors rating categories (superior, satisfactory, needs improvement, failed to attempt, not applicable to session and could not assess in supervision). Overall, 88% of competencies were rated “satisfactory” or “superior” on the WHO Group IPT Task Checklist. Previously trained facilitators consistently scored higher than newly trained facilitators, particularly during pre-group sessions. Across groups, rapport, alliance, and structural adherence were strong, while skills requiring greater clinical judgment, such as emotional exploration, case formulation depth, and role-transition work, initially required improvement. Fidelity ratings improved over time, with “needs improvement” scores decreasing between groups, indicating a clear supervision-driven learning effect. Because fidelity assessment was conducted primarily to support implementation learning rather than formal psychometric evaluation, interrater reliability statistics were not calculated in this pilot phase.

### 3.2 Acceptability and feasibility: qualitative interpretation using the COM-B framework

This section's findings are based on qualitative and implementation data sources, including key informant interviews, focus group discussions with participants and facilitators, structured session observations by the researcher, and supervisors, participant feedback, attendance records, and the WHO Group IPT tools (14). The COM-B model of Capacity, Opportunity, Motivation to Behavior was used because interventions grounded in a theoretical framework have been shown to be more effective than those without in organizing and interpreting implementation experiences and behavioral processes observed during delivery (28, 29). Participant and facilitator experiences were coded and organized according to COM-B domains to support interpretation of how capacity, opportunity, and motivation appeared to influence engagement, intervention delivery, and behavioral participation.

The COM-B model was used as a framework-informed qualitative approach to organize and interpret participants' and facilitators' experiences. Importantly, this study did not measure COM-B constructs using validated quantitative instruments, as the primary aim of this pilot study was to assess the acceptability, feasibility, and implementation processes rather than testing the behavioral mechanism. Therefore, COM-B findings should be interpreted as an explanatory and analytic framework rather than evidence of empirically demonstrated mechanisms of change.

### 3.3 Psychological capability

For the adolescents, the shift in psychological capacity was visible across sessions. In the early meetings, participants found it hard to name what they were feeling or to see any connection between their mood and their relationships. By the middle sessions, adolescents demonstrated increasing understanding of IPT concepts and skills, including linking mood to interpersonal events, emotional expression, and communication strategies. Engagement improved over sessions, particularly in role-plays and problem-solving activities. One participant shared that:

“I was initially anxious when I was told the intervention would be conducted in a group format, but after attending session 1, I realized that I am not alone and that many of my peers are also struggling just like me. This gave me the courage to share and participate in the group sessions”.

Among the facilitators, the development looked different; capability increased through blended training, direct observation, and weekly supervision, narrowing initial skill gaps between newly and previously trained facilitators. The newly trained CHPs began with limited confidence in organizing sessions, delivering psychoeducation, and using the tools in practice.

One described her confusion about the PHQ-9 before the pilot:

“This PHQ-9 we were taught during the training, I thought it was a procedure. I wondered how I was going to do it and thought it was something hard. During the pilot, when we were expected to put what we learned in practice, I realized it was a simple tool to use for assessing depressive symptoms”.

The pairing of more experienced facilitators with a newer one in Group 1, then having that newer facilitator step up to lead Group 2, worked as intended. One incident during the pilot showed how emotionally demanding this kind of work can be. A newly trained male facilitator found himself drawn into a participant's story because it echoed his own past:

“As I was facilitating, she reminded me of my childhood. I went through a similar experience, and that's why I do my best to find solutions for people, especially adolescents. If I don't, I overthink about it and get such headaches.”

Supervisors used the post-session debrief to address this directly, working through the difference between empathy and sympathy, and helping the facilitator see how to stay present without losing his professionalism. As he continued facilitating, he reported a clearer sense of how to distinguish between empathy and sympathy.

### 3.4 Physical capability and opportunity

The intervention was conducted within an existing primary care facility without disrupting normal clinic operations. Sessions were timed during less busy days in the week, Thursday or Friday afternoons, and Saturday mornings, and were held during the school holidays to maximize availability. The IPT-G-A arm retained all 16 participants (100%) on session termination, compared with 11 participants (69%) in the eTAU arm. The familiar setting helped both the facilitators and participants to comfortably participate in sessions. One CHP described the practical benefit:

“Running the sessions in the health facility made it easier for participants to attend, and it allowed us to continue our work as usual and coordinate with other health services when needed.”

A supervisor captured the group's common starting point, and the initial challenges were normalized as a natural part of the learning process rather than disengagement:

“Of course, we've taken them through the theory, the physical, and the virtuals. But practical is usually very different. And we saw a lot of confusion there. We were all confused at first when we started the 1st group. I remember one of the facilitators was drenched in sweat on her 1st pre group session, flipping pages of the IPT-G-A tools throughout the session.”

In spite of these initial difficulties, facilitators showed a steady desire to grow, as a supervisor confirmed:

“You did a good job. What I love about you is that you are willing to learn. You are not afraid of taking up a challenge, you are very okay with that, and open to feedback.”

That gap was, in its own way, useful. It gave the newer facilitators a concrete model of what confident, prepared facilitation looked like, something to watch and work toward over the course of the pilot.

### 3.5 Social opportunity

The group structure offered something that individual therapy in this setting could not readily replicate: a shared space where adolescents gradually recognized themselves in one another. By the second session, the shift was visible. Participants who had been reluctant to speak began to open up; those who felt like they were the only ones struggling with problems found that others were carrying a similar burden. One participant shared:

“It was relieving to hear that other adolescents my age were suffering as well and experiencing similar challenges. The group sessions helped me learn from other participants, and that, really helped.”

The facility's familiarity to both participants and their caregivers, as well as to the facilitators, made it easy for the intervention to progress smoothly. Facilitators based within the facility could flag concerns to colleagues, coordinate referrals, and draw on the broader clinical team without any special arrangements. The collaborative care structure was not a separate system bolted on; it was already there.

### 3.6 Motivation

Participants' attendance and sustained skills practice were enhanced by the following factors: the intervention's relevance, its applicability to real-world interpersonal challenges, and symptom improvement. In addition, continuous supervision and post-session feedback increased facilitators' confidence and commitment to adhering to the structured IPT approach over time.

A participant from Group 2 reflected on how the group learning had given him a new sense of agency:

“I am learning from other people. I am learning from myself. It's a great experience”.

One facilitator observed during a feedback session that the role-play exercises were particularly effective in helping participants apply communication skills to real-life situations:

“Did you see how they opened up and everyone was like, even me. So I think if you look at the examples, then you get to understand better.”

A supervisor noted that participants who actively engaged in group exercises demonstrated the most visible improvements in interpersonal functioning.

One participant described a shift in his relationship with his father:

“I managed to talk to my father, and from that onward, we have a better relationship, and he has stopped drinking alcohol.”

Another, who had suicidal thoughts, reflected on what had shifted:

“After attending these sessions, I have never been suicidal again. I have hope in life now, and I wish to become a doctor.”

The changes reached into households in ways the study had not set out to measure. One mother, whose daughter had presented with severe depression and a recent suicide attempt, and was one of two participants prescribed fluoxetine alongside IPT-G-A, described what the intervention had meant to her:

“I used to think my daughter was okay till when she started attending the sessions and opened up to me. This intervention has opened my eyes and helped my child. I don't think she would have been alive by now if it were not for the intervention. I encourage her to continue with treatment as it has helped her a lot.”

Among the facilitators, motivation manifested differently. The anxiety that characterized the initial sessions, the sweaty, stressed image, gradually transformed into a sense of ownership. They started arriving early, preparing for the sessions, and handling documentation proactively. This transformation was reflected in a small but meaningful moment. When a research assistant moved to take participant files at the end of a session, a facilitator intervened:

“Please do not go with the participants’ files, as we have not finished documenting this current session. We will keep them confidential and handle the files with care and bring them during the next session.”

By the end of the pilot, the facilitators were no longer just delivering the sessions, they were running them.

### 3.7 Behaviour

The combined effect of capability, opportunity, and motivation led to observable behaviors related to intervention adherence (24) that included:

- Consistent session delivery across all treatment phases.
- Completion of weekly symptom ratings by the facilitators.
- Active participation in communication analysis, role-play, and completion of homework by participants.
- Progressive improvement in facilitator competence, skills, and confidence.
- High retention and session completion rates.
- Increased group participation, even among those who were initially anxious.
- Self-care improvement was seen as the participants attended the sessions.

### 3.8 Skill generalization and real-world impact

At the 2-month follow-up, adolescents reported actively applying IPT-G-A skills, including emotion regulation, communication, and problem-solving, to manage school stress, peer conflict, and family tensions. Participants described improved relationships with caregivers, reduced family conflict, greater personal responsibility, and increased mood stability, self-esteem, and hope for the future. Improved communication, particularly a shift from reactive to reflective interaction, was frequently cited as transforming previously strained family relationships, consistent with IPT-G-A role transition and communication analysis components. Reports from adolescents, caregivers, and facilitators consistently indicated that core therapeutic skills generalized to real-world contexts, supporting the potential for meaningful change following the brief *Ushirikiano intervention* in primary care settings.

### 3.9 Quantitative results for the Ushirikiano treatment model

Quantitative outcomes are presented descriptively because this pilot randomized controlled trial was designed to evaluate feasibility, acceptability, and implementation procedures, and to generate estimates to inform a future fully powered trial, rather than to establish treatment effectiveness. Descriptive statistics are reported using means, proportions, and observed trends over time. Given the small sample size (*n* = 32), formal hypothesis testing was not undertaken, and effect estimates should be interpreted cautiously.

#### 3.9.1 Baseline demographics and clinical characteristics of participants for the pilot study

A total of 155 participants were screened for eligibility, of whom 36 met diagnostic criteria for at least one mental disorder. Of the 36 eligible participants, 32 were enrolled and randomized equally to IPT-G-A (*n* = 16) and enhanced treatment as usual (eTAU) (*n* = 16).

Baseline demographic and clinical characteristics are presented in Table 2. The baseline characteristics were broadly comparable between study arms with respect to age, sex distribution, educational status, clinical presentation, and baseline functional impairment. Participants had a mean age of approximately 15.6 years and equal representation of females (*n* = 16, 50%) and males (*n* = 16, 50%) across the arms. Most participants enrolled were in secondary school (Form 2–4: *n* = 16), with a smaller proportion attending upper primary school (*n* = 13) or having discontinued formal education (*n* = 1).

**Table 2:** Baseline demographic and clinical characteristics of participants by study Arm.

Characteristic	IPT-G-A (*n* = 16)	eTAU (*n* = 16)	Total (*n* = 32)
Demographic characteristics			
Age, mean (years)^a^	15.8	15.3	15.6
Female, *n* (%)	8 (50.0)	8 (50.0)	16 (50.0)
Male, *n* (%)	8 (50.0)	8 (50.0)	16 (50.0)
School attendance, *n* (%)			
Completed form 4 (Highschool)	2 (12.5)	0 (0.0)	2 (6.3)
Form 3	3 (18.8)	5 (31.3)	8 (25.0)
Form 2	5 (31.3)	3 (18.8)	8 (25.0)
Grade 9	1 (6.3)	3 (18.8)	4 (12.5)
Grade 8	1 (6.3)	3 (18.8)	4 (12.5)
Grade 7	3 (18.8)	2 (12.5)	5 (15.6)
Drop out	1 (6.3)	0 (0.0)	1 (3.1)
Clinical characteristics, *n* (%):			
Major Depressive Disorder (MDD), Generalized Anxiety Disorder (GAD), Somatic Symptom Disorder (SSD)			
MDD—Severe + suicidal ideations	5 (31.3)	3 (18.8)	8 (25.0)
MDD—Moderate	3 (18.8)	1 (6.3)	4 (12.5)
MDD—Mild	2 (12.5)	0 (0.0)	2 (6.3)
MDD—Severe + suicidal ideations + GAD	1 (6.3)	4 (25.0)	5 (15.6)
MDD—Severe−suicidal ideations + GAD	0 (0.0)	1 (6.3)	1 (3.1)
MDD—Moderate + GAD	0 (0.0)	1 (6.3)	1 (3.1)
SSD + MDD—Severe + suicidal ideations	3 (18.8)	2 (12.5)	5 (15.6)
SSD + MDD—Severe—suicidal ideations	0 (0.0)	1 (6.3)	1 (3.1)
SSD + MDD—Severe + suicidal ideations + GAD	1 (6.3)	0 (0.0)	1 (3.1)
SSD + MDD—Mild	1 (6.3)	1 (6.3)	2 (6.3)
SSD	0 (0.0)	2 (12.5)	2 (6.3)
Medication status, *n* (%)			
IPT-G-A with Fluoxetine 20 mg daily	2 (12.5)	0 (0.0)	2 (6.3)
Functional Impairment:			
WHO DAS 2.0 scores (0: None, 1: Mild, 2: Moderate, 3: Severe, 4: Extreme or cannot do)^b^			
S1: Standing for long periods such as 30 min	1	1	1
S2 Taking care of your household responsibilities	0	1	1
S3 Learning a new task (e.g., how to get to a new place)	1	2	2
S4 Joining in community activities in the same way as anyone else	2	2	2
S5 How much have you been emotionally affected by your health problems	2	2	2
S6 Concentrating on doing something for ten minutes	2	2	2
S7 Walking a long distance such as a kilometer	1	2	2
S8: Washing your whole body & S9: Getting dressed	0	0	0
S10: Dealing with people you do not know	3	2	3
S11: Maintaining a friendship	2	1	2
S12: Your day-to-day work	1	1	1

a Standard deviation (SD) not reported because variability estimates were not calculated for this pilot analysis (0: None, 1: Mild, 2: Moderate, 3: Severe, 4: Extreme or cannot do), not participant frequencies.

b WHO DAS 2.0 values represented average functional impairment severity score.

Major depressive disorder (MDD) was the predominant diagnosis (*n* = 17, 53.1%) across both study arms. Several adolescents presented with co-occurring generalized anxiety (GAD; *n* = 7, 21.9%) and/or somatic symptom disorder (SSD; *n* = 6, 18.8%), reflecting the complex clinical presentation of emotional distress in this population.

Baseline depressive symptom severity was generally high, with many participants presenting with severe depressive symptoms and suicidal ideation. Across both study arms, severe MDD with suicidal ideation represented the largest clinical subgroup. Only two participants (both in the IPT-G-A arm) received adjunctive fluoxetine due to severe depression accompanied by active suicidal ideation and prior suicidal attempts. This decision was made after consultations between the psychiatrist, psychologists, and the IPT-G supervisors.

Baseline WHO DAS 2.0 findings suggested moderate impairment across emotional, cognitive, and social functioning domains, particularly in concentration, community participation, maintaining relationships, and adaptation to new tasks. Self-care impairment remained unaffected across the participants. As expected in a pilot feasibility trial, baseline analyses were descriptive and intended to characterize the sample and assess balance between study arms rather than test statistical equivalence.

#### 3.9.2 Preliminary effectiveness of the Ushirikiano treatment model

**Figure 3:**
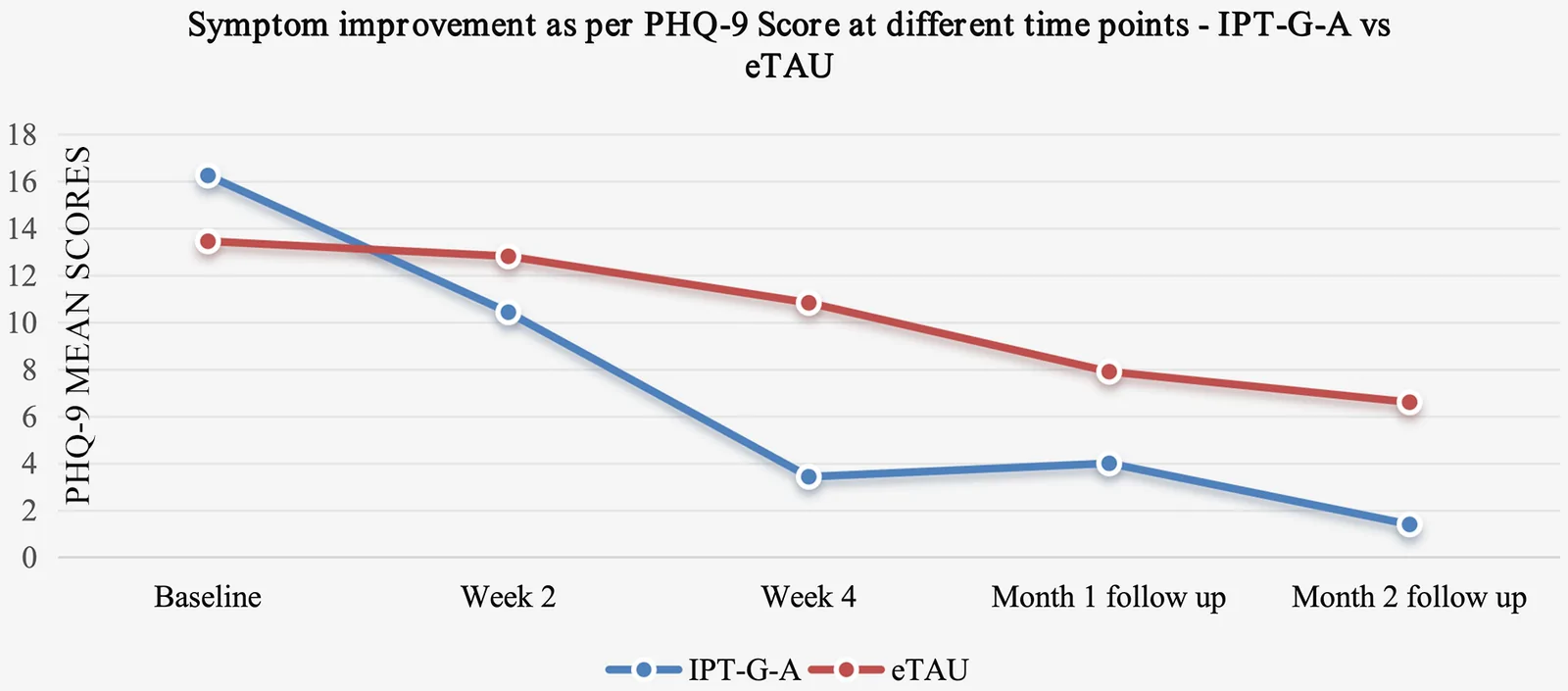
Mean PHQ-9 scores at baseline, week 2, week 4, month 1, and month 2 follow-up for IPT-G- A and enhanced treatment as usual (eTAU).

**Figure 4:**
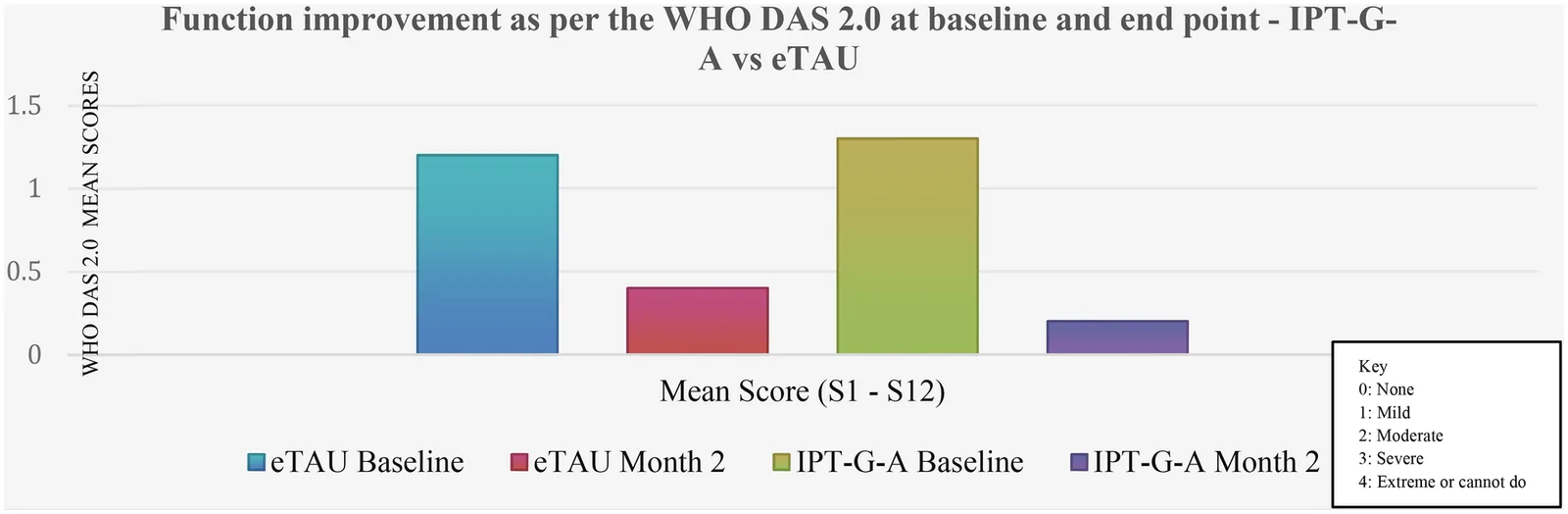
Mean wHO-DAS 2.0 domain scores at baseline, and month 2 follow-up for enhanced treatment as usual (eTAU), and IPT-G-A. Higher scores indicate greater functional impairment.

The pilot results indicated a faster potential decline in depressive symptoms in the IPT-G-A group compared to eTAU. At the 2-month follow-up, all but one IPT-G-A participant (who had moderate severity) reached symptomatic remission. In contrast, only one eTAU participant did, with most experiencing mild symptoms and two with moderate or moderate-to-severe severity (Figure 3). Functional outcomes from this pilot study favored IPT-G-A across several WHO-DAS 2.0 domains, with gains maintained at month 2 follow-up (Figure 4). These findings should be interpreted with caution because the primary purpose of the randomized controlled trial was to assess the acceptability, feasibility, and implementation procedure of the Ushirikiano treatment model rather than to establish treatment efficacy. The study was not powered to detect statistically significant differences between groups; therefore, the observed improvements should be considered preliminary indications of potential benefit. Moreover, this pilot study did not assess outcomes for anxiety disorder or somatic symptom disorder; these additional two disorders will be evaluated in the planned fully powered RCT.

Only 2 participants required augmentation with fluoxetine 20 mg daily, as they both had severe depression with active suicidal ideation and had attempted suicide a few times. This decision followed consultation among the research team, which included a psychiatrist, psychologists, and IPT supervisors. The rest of the participants who also had severe depression with suicidal ideation or attempts were monitored closely during the intervention, the same as those in the eTAU. The participants on medication used it for 2 months and felt they could manage with what they had learned in the IPT-G-A sessions. Augmentation of an antidepressant with IPT-G-A seemed to facilitate quicker improvement in their depressive symptoms, though this result should be interpreted with caution due to the small number who used fluoxetine and the study's pilot nature.

## 4 Discussions

This pilot randomized controlled trial provides early evidence that the *Ushirikiano treatment model* is acceptable and feasible in a low-resource urban primary care setting in Kenya. Most enrolled adolescents presented with severe major depressive disorder, frequently with comorbid somatic symptoms, consistent with evidence that depression is a leading contributor to disability among adolescents in sub-Saharan Africa and Kenya, and is often expressed through somatic distress (4, 32, 33). These findings support the need for integrated, biopsychosocial assessment and care. Throughout this Discussion, illustrative quotations from adolescents, caregivers, and facilitators are presented to characterize participants’ subjective experiences of acceptability, engagement, and perceived change, in line with this pilot's focus on feasibility and implementation. These accounts should be read as evidence of how the intervention was experienced and received, not as evidence of clinical effectiveness; the latter is addressed separately, and cautiously, through the descriptive quantitative findings reported above.

### 4.1 Biopsychosocial formulation and IPT problem areas

A major strength of the *Ushirikiano treatment model* is its foundation in IPT's biopsychosocial formulation. The pre-group individual session enabled systematic identification of interpersonal stressors, such as family conflict, parental substance use, violence, and economic hardship, and guided focused intervention during group sessions (14, 34). Interpersonal conflict and role transitions were the most common IPT problem areas, reflecting adolescents' developmental and socioeconomic contexts. This presentation of problem areas is not unusual in the literature; a school-based IPT-G feasibility study in Nepal found exactly the same two areas dominating and identified as the main areas of focus in the group sessions (35). Although social isolation and grief were less frequently identified as primary problem areas, the group format directly addressed isolation, in addition to the commonly experienced IPT problem areas, because isolation was a pervasive feature of the clinical picture across both groups, whereas grief appeared less suited to the condensed four-session format (36).

#### 4.1.1 Interpersonal relationships as a source of emotional distress

Across the two groups involved in the pilot study, participants often described interpersonal relationships as central to their well-being. Conflicts with parents, caregivers, peers, and romantic partners were frequently reported and were often linked to feelings of sadness, frustration, and social isolation. Participants cited misunderstandings, lack of communication, and perceived rejection as key factors contributing to their emotional distress.

“When others shared their stories, I realized I was not the only one going through these things. Hearing that other people also struggle with parents or friends made me feel less alone. After learning about communication in the group, I tried to discuss a concern about my mother's behavior. It helped us understand each other, and she no longer sees other men or brings them home.”

One participant demonstrated deep conceptual understanding of the IPT framework by articulating the interpersonal basis of his depression independently:

“Depression is not coming on its own. It has been triggered by someone or something. There is an interpersonal basis. I feel low because last week, when I went back home, I heard that my mother was asking for financial support because she had spread the word that I was dead.”

Another participant described the emotional weight of feeling like a failure in his key relationships:

“I experienced failure, I felt like I failed, I felt like I failed my family, I felt like I let them down.”

A further participant reflected on the improvement in a previously strained relationship with the brother following the application of communication skills learned in the group:

“We are living together. I couldn't even sit with him. But now I sit with him. He even bought me lunch one day, when we met in town, which made me feel cared for.”

These stories, which recurred throughout interviews with participants and session recordings, demonstrate how the IPT-G-A framework helped adolescents apply group learning to their everyday lives and make it meaningful for interpersonal change. This pattern was verified by facilitators, who reported that as participants began to associate their mood with specific interpersonal events, their engagement increased and therapeutic gains were made in both groups.

#### 4.1.2 Role transition and adolescent stress

Many participants discussed challenges related to developmental changes, including academic pressures, shifting friendships, changing family roles, and caregiver expectations. These changes were often felt as stressful and linked to feelings of uncertainty, inadequacy, or loss of support. Facilitators noticed that discussing these changes within the group helped participants better understand their emotional responses and normalize their experiences.

Participants' outlook on life and relationships improved after attending the sessions. They learned to stop trying to change others and instead to adjust their own approach and responses to various situations.

“To support my academics and provide for my family, I do all sorts of casual jobs, including collecting items at the dumpsite. It used to sadden me that after all my hard work, my mother takes all the money I have made for the day. After the sessions, I changed my perspective towards mom and realized that mom can't change. I now listen quietly when she shouts at me, and later I respond calmly when she has cooled off.”

Facilitators explicitly affirmed role transition experiences during sessions, telling one participant:

“You were a housekeeper, but now you are a provider. Your role has changed, and that's how life is sometimes.”

In the feedback sessions, one supervisor observed that role transition was the most frequently reported area of IPT problem in both groups, and that facilitators gradually became more capable of helping participants explore the losses associated with old roles and the opportunities inherent in new roles. These facilitated reframes helped participants identify their struggles within the IPT framework, reduced self-blame, and fostered adaptive coping responses to the role demands they encountered.

### 4.2 Pharmacological management within the Ushirikiano treatment model

The Ushirikiano treatment approach to pharmacotherapy sits within the framework recommended by the WHO mhGAP Intervention Guide, which identifies antidepressant medication, and specifically fluoxetine, as an appropriate adjunct to psychological care in severe presentations, subject to adequate clinical oversight (6).

Only two participants in this pilot were eligible to receive fluoxetine 20 mg daily in addition to IPT-G-A. Both were psycho-educated, along with their guardians, about the medication, and close monitoring was conducted to assess any side effects. Broader evidence from Kenya supports this multimodal approach, as shown in the SMART-DAPPER trial, a sequential Multiple Assignment Randomized Trial conducted in Kisumu, Kenya, which tested non-specialist-delivered IPT and nurse-prescribed fluoxetine as first-line treatment for adults with Major depressive disorder (MDD) or Post-traumatic stress disorder (PTSD) (19, 37). The qualitative results from the 2024 SMART-DAPPER project indicated that patients reported improvements at individual, interpersonal, and community levels, and found both IPT and fluoxetine acceptable (38). International evidence points in the same direction, as indicated in the 2025 randomized controlled trial in China, where group psychotherapy combined with medication produced a response rate of 97.8% in depressed adolescents, compared with 80% for the medication alone, demonstrating the additive benefit of combining the two approaches in this age group (39). A 2021 RCT in Kenya at an HIV clinic in Kisumu also showed that non-specialists delivered IPT significantly improved MDD and PTSD diagnosis, symptom severity, and disability among HIV positive women affected by gender based violence, relative to waiting list control (20). All in all, these Kenyan studies establish a clear regional evidence base for non-specialist-delivered IPT in primary care settings and provide the precedent on which the Ushirikiano treatment model's adolescent adaptation builds. This regional evidence extends beyond Kenya: earlier cluster-randomized trials of group IPT among adults in rural Uganda (39) and among orphaned and vulnerable adolescents in South Africa (26) support the feasibility of non-specialist-delivered group IPT for depression across diverse sub-Saharan African contexts, beyond the Kenyan studies on which the Ushirikiano model most directly builds. Because two participants in the intervention arm received fluoxetine alongside IPT-G-A, the Ushirikiano model, as piloted, should be understood as a combined psychotherapy-plus-medication package rather than IPT-G-A delivered in isolation. With only two participants on medication, this pilot was not statistically positioned, to disentangle the relative contribution of fluoxetine vs. IPT-G-A to the observed improvements; any apparent additive benefit of medication noted in the Results should therefore be regarded as hypothesis-generating rather than confirmatory, and the planned fully powered trial will need to address this question directly, for example, through pre-specified subgroup or sensitivity analyses by medication status.

### 4.3 Positioning Ushirikiano within broader task-shifted psychological interventions for adolescent depression in LMICs

A 2026 realist systematic review and meta-analysis of group psychosocial interventions for depression, anxiety, and post-traumatic stress disorder among children and adolescents of mixed gender across low- and middle-income countries found that interventions work best when age-appropriate content and activities together with cultural adaptation are incorporated, explaining the substantial heterogeneity found, which is moderated by participant age, social demographic factors, and conflict-affected status (40). Beyond IPT, several other brief, lay-provider-delivered psychological interventions have shown promise for adolescent depression in low-resource settings and provide useful comparators for the Ushirikiano model. In Kenya, a large 5-arm randomized trial conducted during the COVID-19 pandemic found that the Shamiri intervention, which draws on growth-mindset, gratitude, and values-affirmation exercises delivered by recent secondary-school graduates, and its individual components reduced depression and anxiety symptoms among school-going adolescents (41). In India, a brief, transdiagnostic problem-solving intervention delivered by lay school counsellors reduced depressive, anxiety, and conduct-related symptoms among adolescents in low-income urban schools in New Delhi, demonstrating that this type of lay-delivered psychological care can be tailored specifically to school-going adolescent populations rather than adapted from adult primary-care programs (42). School-based group cognitive behavioral therapy delivered by trained teachers has similarly reduced depressive symptoms among adolescents in south-west Nigeria (43), and in Zimbabwe, a cluster-randomized implementation trial comparing community-health-worker-delivered Friendship Bench problem-solving therapy with a peer-delivered Youth Friendship Bench model for adolescent common mental disorders found the youth-led model to be feasible and highly valued, with participants particularly appreciating the ability to connect with a same-age peer, although further optimization was needed to strengthen its clinical and cost-effectiveness (44). Together, these findings indicate that several brief, non-specialist-delivered psychological approaches are feasible and, in many cases, effective for youth depression and anxiety across sub-Saharan Africa and South Asia, positioning group IPT-A as one of several promising, scalable options for this population rather than the only evidence-based approach.

### 4.4 Acceptability, mixed-gender groups, and retention

The 100% retention in the Ushirikiano arm, compared with 69% in the eTAU, was the most straightforward indicator of feasibility in the pilot. Mixed-gender groups were operationally workable, though they required deliberate facilitation; female, younger, and anxious participants needed active encouragement in early sessions (1, 10, 36), while placing siblings or cousins/relatives in the same group restricted disclosure by at least one participant, resulting in limited benefit from the intervention. The full-powered trial will build in a systematic protocol for separating family members across groups, with confidentiality reinforced at contracting and revised in all sessions.

The retention finding is consistent with Yator et al.'s work in Kenya, which showed that community health promoters delivering IPT-G was acceptable and feasible for depressed adolescent mothers living with HIV (25). The INSPIRE feasibility trial in Nairobi similarly found that combining mhGAP and adapted IPT-G was appropriate for integration into routine primary care for pregnant adolescents (24).

The group format encouraged interpersonal learning and skill growth through activities such as communication analysis, role-play, homework, and problem-solving exercises. These methods allowed participants to reflect on their interactions and improve their communication strategies. Facilitators observed that these activities offered insights into participants' interpersonal patterns and helped them develop new responses to relationship challenges. A participant's reflection confirmed this observation.

“Sometimes someone else in the group would suggest another way of handling a situation. That helped me think differently about my own problems.”

Emotional processing evolved throughout the intervention. Early sessions were marked by hesitation and limited self-disclosure. However, as trust within the group grew and participants realized they were not alone in their struggles, they became more willing to share personal experiences and express emotions. Peer support played a crucial role in this progress, with participants validating each other's experiences, offering encouragement, and suggesting solutions. A facilitator shared her observation:

“During the first sessions, many participants were quiet and hesitant, and some would constantly look down. But as the group progressed, they became more comfortable sharing personal experiences and discussing how their relationships affected their mood.”

One participant described the moment she realized the group was a safe space for honest disclosure:

“It's okay if this is a safe space, I’ll try opening up.”

One participant described the shift from isolation to connection that occurred as the sessions progressed:

“I was worried that I wouldn't be able to speak. But when I came here, I spoke out and gave some solutions.”

A further participant captured the emotional relief that came from realizing others shared similar struggles:

“We are giving hope. Because hope is the most important thing in life.”

This development of reluctance to active emotional expression was similar across both groups and was directly correlated with improvements in PHQ-9 scores. Emotional expressiveness was also a fundamental process of therapeutic change in the IPT-G-A model, with participants who exhibited the most emotional opening also showing the most rapid symptom reductions by session 4 (termination).

### 4.5 Facilitator fidelity and supervision

Fidelity data showed steady improvement in formulation depth and IPT technique over the pilot, underscoring the critical role of structured supervision (45, 46). Facilitators reported continuous supervision and fidelity checks improved their effectiveness in delivering the intervention. Feedback from supervisors helped them refine skills such as linking emotions to interpersonal events, fostering emotional exploration, and maintaining focus on IPT-problem areas. Over time, facilitators gained more confidence in managing group dynamics and motivating participant involvement.

“The manual helped guide us through each session. Having a clear structure made it easier to facilitate discussions and keep the group focused. In the beginning, it was challenging to help participants explore their emotions more deeply. But with supervision and practice, we became more confident in guiding those conversations.”

Another supervisor reflected on the learning trajectory observed across the pilot:

“This is piloting. So, piloting means you need to learn and make all your mistakes and bring them to our discussion. No one is perfect. We all start from somewhere.”

One facilitator described the supervision process as essential to managing the emotional demands of facilitation:

“These sessions are not very heavy on you when you're doing it. But afterward, all the negativity, you absorb all of them — those negative energies from eight different people — without you knowing, you're taking it.”

A 2024 global content analysis of IPT implementation across 31 countries concluded that IPT is relatively straightforward to train non-specialists, and is broadly perceived as culturally congruent, including in relational cultures such as Kenya, where the interpersonal framework maps naturally onto communal patterns of distress and help-seeking (45).

The supervision model in this pilot also carried the emotional weight of the work. Sessions with severely depressed or suicidal adolescents who had experienced difficult life circumstances generated secondary stress for facilitators, and the countertransference incident described in the results underscores how quickly personal material can surface. A supervisor noted the importance of self-care and emotional debriefing as part of the supervision model:

“You need to learn how to detach. And detachment doesn't come all at once. Give yourself time to love yourself a bit. For me to be okay and for me to help other people.”

The structured weekly feedback model, combined with real-time observation and reflective debriefing, created a learning environment in which facilitators grew in competence over time, gained better control of their emotional reactions, and became more confident and faithful in delivering the intervention across the four group sessions. Facilitator well-being, self-care protocols, and vicarious trauma support need to be built into the supervision structure of any future trial as a core component, not an optional extra.

### 4.6 Preliminary outcomes and clinical significance

Preliminary outcomes indicated potential for faster, more complete reductions in depressive symptoms and greater functional improvements across emotional, cognitive, and social domains among IPT-G-A participants compared with eTAU. These gains, alongside reported real-world application of skills, indicate strong clinical potential. These findings are supported by Kenyan studies on IPT, which are comparable to or exceed those observed in physician-delivered care in high-income settings (19, 39, 47). It is important to note that augmenting fluoxetine for the two participants in the IPT-G-A arm seemed to yield a quicker response to the combined treatment (IPT-G-A & fluoxetine) than for those who received IPT-G-A only or those in the eTAU arm. Despite the small number, the exclusive use of medication within the intervention arm may have acted as a confounder, contributing to the greater symptom improvement observed relative to eTAU. Despite the small number of participants who received both IPT-G-A and fluoxetine, the improvement seen in this pilot cannot be attributed to IPT-G-A alone, though it is premature to conclude that the effect observed is due to the intervention provided. Preliminary functional gains on the WHO-DAS 2.0 were stable or continued to improve at the month 2 follow-up, rather than fading, for most adolescents after treatment ended, potentially suggesting that the brief format was useful for initiating symptom relief.

### 4.7 Limitations

Despite limitations, including small sample size, single-site design, and short follow-up, these findings support progression to a fully powered randomized controlled trial. In our future studies, we would need to strengthen formulation-focused training, refine group composition strategies, incorporate facilitator well-being into supervision, and extend follow-up. Other groups doing similar efforts have incorporated cost-effectiveness into their work as well (19, 46, 48, 49). Overall, the Ushirikiano model offers a promising, scalable approach to addressing adolescent mental health needs in resource-constrained primary care settings.

## 5 Conclusion

The Ushirikiano treatment model brings together task-shifted IPT-G-A and medication, when clinically appropriate, all within existing community health infrastructure. The pilot showed that the intervention can be delivered reliably and is accepted by adolescents, guardians, and facilitators dealing with often severe major depressive disorder. It leads to early improvements in functioning and symptoms, although the long-term durability of these benefits needs further assessment in future studies with extended follow-up. Designed for low-resource settings, and supported by growing regional evidence from Kenya and East Africa, these findings reasonably support scaling up the intervention to include assessment of all three common mental disorders (Major depressive disorder, anxiety disorder, and somatic symptom disorder).

## Data Availability

The original contributions presented in the study are included in the article/Supplementary Material, further inquiries can be directed to the corresponding author/s.
